# Cellular senescence-related gene signature as a valuable predictor of prognosis in hepatocellular carcinoma

**DOI:** 10.18632/aging.204658

**Published:** 2023-04-13

**Authors:** Shuqiao Zhang, Yilu Zheng, Xinyu Li, Shijun Zhang, Hao Hu, Weihong Kuang

**Affiliations:** 1 First Affiliated Hospital of Guangzhou University of Chinese Medicine, Guangzhou University of Chinese Medicine, Guangzhou, Guangdong, China; 2 Department of Hematology, The Seventh Affiliated Hospital, Sun Yat-sen University, Guangzhou, Guangdong, China; 3 Medical College of Acupuncture-Moxibustion and Rehabilitation, Guangzhou University of Chinese Medicine, Guangzhou, Guangdong, China; 4 Department of Traditional Chinese Medicine, The First Affiliated Hospital, Sun Yat-sen University, Guangzhou, Guangdong, China; 5 Guangdong Key Laboratory for Research and Development of Natural Drugs, School of Pharmacy, The First Dongguan Affiliated Hospital of Guangdong Medical University, Guangdong Medical University, Dongguan, Guangdong, China

**Keywords:** hepatocellular carcinoma, cellular senescence, prognosis, immune, machine learning

## Abstract

Background: Hepatocellular carcinoma (HCC) is a lethal tumor. Its prognosis prediction remains a challenge. Meanwhile, cellular senescence, one of the hallmarks of cancer, and its related prognostic genes signature can provide critical information for clinical decision-making.

Method: Using bulk RNA sequencing and microarray data of HCC samples, we established a senescence score model via multi-machine learning algorithms to predict the prognosis of HCC. Single-cell and pseudo-time trajectory analyses were used to explore the hub genes of the senescence score model in HCC sample differentiation.

Result: A machine learning model based on cellular senescence gene expression profiles was identified in predicting HCC prognosis. The feasibility and accuracy of the senescence score model were confirmed in external validation and comparison with other models. Moreover, we analyzed the immune response, immune checkpoints, and sensitivity to immunotherapy drugs of HCC patients in different prognostic risk groups. Pseudo-time analyses identified four hub genes in HCC progression, including CDCA8, CENPA, SPC25, and TTK, and indicated related cellular senescence.

Conclusions: This study identified a prognostic model of HCC by cellular senescence-related gene expression and insight into novel potential targeted therapies.

## INTRODUCTION

Hepatocellular carcinoma (HCC) is lethal cancer, with approximately 1 million people diagnosed in 2020 [1, 2]. Chronic alcohol consumption, diabetes or obesity, metabolic syndromes, and infection by the hepatitis B virus are vital factors responsible for HCC progression, which promotes cirrhosis, ultimately HCC [3, 4]. Patients with early-stage HCC are frequently asymptomatic, significantly delaying diagnosis and contributing to neoplastic. Effective treatment options are extremely limited in the advanced stages of definitively diagnosed HCC [5]. In addition, a significant number of experiments revealed that the dysregulated telomere maintenance, chromatin modification, cell cycle system, and oxidative stress in HCC cells, as well as extensive mutation or abnormal gene expression, reduce the effectiveness of targeted medications [6, 7]. Although more and more gene signatures are being investigated to guide therapy, the prognosis prediction of HCC patients is less satisfactory. Therefore, innovative biomarkers are urgently needed to guide clinical decision-making to discern people with a high risk of HCC.

Over the past decade, research elucidated that cellular senescence, a fundamental hallmark of cancer, is closely linked to typical carcinogenesis, tumor development, and cancer cell invasiveness of HCC [8–10]. Cellular senescence is a way to the irreversible cessation of cell proliferation [11]. Multiple therapeutic therapies cause senescence in cancer cells by inducing genotoxic stress, hyperactivation of mitogenic signaling, or oxidative stress, resulting in a stable cell cycle halt [12]. Therefore, therapy-induced senescence is an initial antitumor strategy to halt proliferation and prevent additional genomic instability.

Despite substantial research, the role of cellular senescence in HCC remains unclear. A systematic evaluation of the prognostic signature of cellular senescence in HCC patients could advance our understanding of the mechanisms underlying HCC and provide novel approaches for accurate diagnosis and treatments. Gene signatures derived from machine learning can help assess cancer prognosis and steer immunotherapy [13]. This study comprehensively analyzed the prognostic-related gene expression data and corresponding HCC clinical information. Ultimately, we determined a cellular senescence score genes model that could serve as a prognostic predictor for patients with HCC.

## MATERIALS AND METHODS

### Data collection

From the TCGA database (https://portal.gdc.cancer.gov/repository), we retrieved the RNA sequencing data of 377 patients, and 365 patients were chosen for subsequent analysis after 12 patients with missing data on survival status or gene expression data were excluded. Additionally, 231 patients’ RNA-seq data with prognostic data were extracted from the HCCDB (http://lifeome.net/database/hccdb) database. TCGA-LIHC (Liver hepatocellular carcinoma) and HCCDB18 (Liver hepatocellular carcinoma-Japan) patients’ clinical data are displayed in Supplementary Table 1. Two bulk transcript datasets (GSE121248, GSE45267) were collected from GEO database (https://www.ncbi.nlm.nih.gov/geo/), which included tumor and normal human liver tissues (Supplementary Tables 2, 3). Genotype-Tissue Expression (GTEx) databases (https://gtexportal.org/home/) were queried for gene expression information on 110 normal liver samples (Supplementary Table 4). The same sequencing platform treated the gene expression data in GTEx databases as the TCGA database to minimize potential batch effects. Based on this, we merged gene expression data from TCGA-LIHC and GTEx using the “combat” function of the “sva” package in R software. The combat function in the “sva” is a classical Bayes-based analysis that applies known batch information to batch-correct a normalized high-throughput data matrix from TCGA-LIHC and GTEx and then outputs a batch-corrected merged data matrix. 1582 genes involved in cellular senescence were collected from the Molecular Signatures database (http://www.gsea-msigdb.org/) and cell senescence database (https://genomics.senescence.info/cells/) (Supplementary Table 5). Expression data in all datasets were normalized by log2 (FPKM+1).

### Variance analysis

The cellular senescence-related gene in normal and HCC samples from TCGA-LIHC and GTEx datasets was assessed using the Wilcoxon test. The gene was considered significant if the false discovery rate was < 0.05 and |logFC| > 1.00. Meanwhile, two datasets performed weight gene co-expression network analysis (WGCNA) on the gene. In parallel, WGCNA was performed on the genes in the merged dataset. The soft-power parameters ranging from 1 to 20 were evaluated based on the scale-free topology criterion. Optimal values were selected to convert the correlation matrix into an adjacency matrix and then into a topological overlap matrix. The minimum module size was set to 50 using the average-linkage hierarchical clustering approach to cluster genes based on the topological overlap matrix (TOM). Following this, related modules were merged. A Pearson correlation test determined the association between integrated modules with tumor and non-tumor specimen types. Finally, the genes that resulted from the intersection of the Wilcoxon test and the WGCNA analysis were considered cellular senescence-related genes (DEGs) for further research.

### Gene ontology and KEGG analysis

In order to shed further light on the biological processes (BP), cellular components (CC), molecular functions (MF), and pathways involved with DEGs in HCC. The R “clusterProfiler” tool analyzed cellular senescence-related DEGs in HCC using Gene Ontology (GO) and the Kyoto Encyclopedia of Genes and Genomes (KEGG).

### Gene signature construction using multiple machine learning algorithms

First, univariate and multivariate cox regression analysis identified cellular senescence-related DEGs with prognosis value. Only those DEGs with P < 0.05 were used for subsequent constructs. SVM-RFE uses the “e1071” and “msvmRFE” SVM modeling packages to find the optimal gene by eliminating feature vectors [14]. In the meantime, we used the Random Forest (RF) algorithm [15] to select genes with significant clinical survival variables. Then the core genes in the intersection of RF and SVM-REF results were penalized using LASSO Cox regression to identify the cellular senescence-related genes signature for a more refined model and calculate the coefficient of each gene in the signature. Finally, cellular senescence associated genes signature was developed and defined as a senescence score model the formula of the senescence score model: senescence score $=\;{\text{Σ}}_{i}^{19}x_{i}\;\times \;y_{i}\;(X\;:\;\text{coefficients},\;Y\;:\;\text{gene}\;\text{expression}\;\text{level})$. The median senescence score stratifies HCC patients into low-risk and high-risk subgroups with different prognostic situations. The cBioPortal’s (https://www.cbioportal.org) standard processing pipeline assessed the mutation profiles of predicted genes derived from the preceding steps. Cellular senescence-related genes signature was also analyzed by MCODE [16] and Metascape (https://metascape.org) [17].

### Immune status analysis

To analyze significant biological pathways of different subgroups, GSEA [18] was utilized. Immune scores and immune cell infiltration levels of HCC patients in different subgroups were assessed using single-sample gene set enrichment analysis (ssGSEA) [7, 19]. The algorithms for evaluating the association of the two risk groups with cellular immune responses involved CIBERSORT [20], CIBERSORT−ABS [21], QUANTISEQ [22], MCPCOUNTER [23], XCELL [24], EPIC [25], and TIMER [26]. The immune functions in subgroups were further compared. In addition, we predicted drug sensitivity in tumor samples by cell line expression profiling. The “oncoPredict” R tool predicted the patients’ half-maximal inhibitory concentration (IC50) of drugs using ridge regression and 10-fold cross-validation.

### Single-cell trajectory analysis

The hub genes of the predictive model were evaluated in HCC samples using single-cell trajectory analysis. Raw single-cell transcriptome profiling data for ten HCC patients from two relevant sites, primary tumor (HCC01T, HCC02T, HCC03T, HCC04T, HCC05T, HCC06T, HCC07T, HCC08T, HCC09T, and HCC10T) and non-tumor liver (HCC03N, HCC04N, HCC05N, HCC06N, HCC07N, HCC08N, HCC09N, and HCC10N), was achieved from GEO (GSE149614) dataset. We used “Seurat” and “Monocle” packages in R to process the data. Gene number, relative hemoglobin, and mitochondrial and ribosomal abundance (Supplementary Figure 1A, 1B), indicating that the cellular readouts were comparable between samples and no transcriptional batch effects were observed. Cells with <2500 or > 20000 detected genes containing mitochondrial genome > 4% were excluded. Next, single-cell data were normalized, and variable genes were hunted by the “SCTransform” method. The “SCTransform” method models single-cell unique molecular identifiers expression data using regularized negative binomial regression to remove variation due to sequencing depth. After, 20 most powerful principal components were found by PCA analysis (Supplementary Figure 2). Further dimension reduction of those principal components was proceeded by the UMAP method to visualize cell distribution. Cell types of principal components were annotated by “CellMarker” (http://xteam.xbio.top/CellMarker/) and “PanglaoDB” (https://panglaodb.se/) databases. Then cells in HCC specimens were split into the different state by Pseudo-time analysis using the monocle algorithm [27].

### Statistical analysis

The analyses were all employed by R 4.0.5 software. Kaplan Meier (KM) analysis were conducted by “ggsurvplot” package. The time-dependent receiver operator characteristic curves (ROC) and decision curves analysis (DCA) were generated using “timeRoc” and “ggDCA” packages [28]. The Benjamin Hochberg approach was used to minimize the false-positive rate of differential gene expression. Analyses used two-tailed p-values <0.05.

### Availability of data and materials

The data and materials supporting this study’s findings are available from the corresponding author upon reasonable request.

## RESULTS

### Acquisition of DEGs

**Figure 1:**
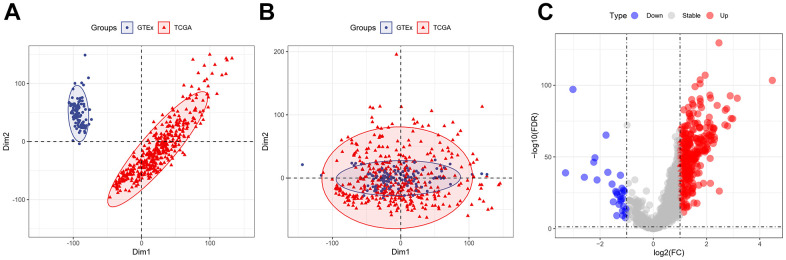
**Expression of the cellular senescence-related genes.** (**A**) PCA analysis of gene expression profiles from TCGA-LIHC and GTEx datasets before batch effect removal. (**B**) After batch effect removal, PCA analysis of gene expression derived from two different datasets. (**C**) The volcano plots of the combined dataset’s cellular senescence-related genes.

**Figure 2:**
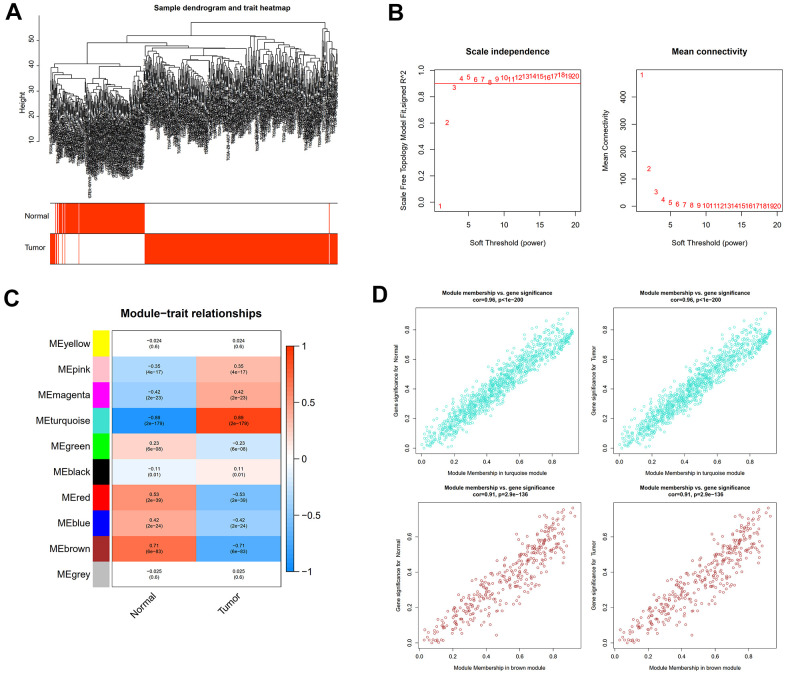
**WGCNA analysis to uncover the key pathogenic module.** (**A**) Sample clustering tree with pathological state. (**B**) The determination of the power of the soft threshold for the combined dataset. (**C**) A heatmap depicting the relationship between module eigengenes and liver pathology. (**D**) Scatter plots illustrate the genes’ significance versus membership in brown and turquoise modules.

**Figure 3:**
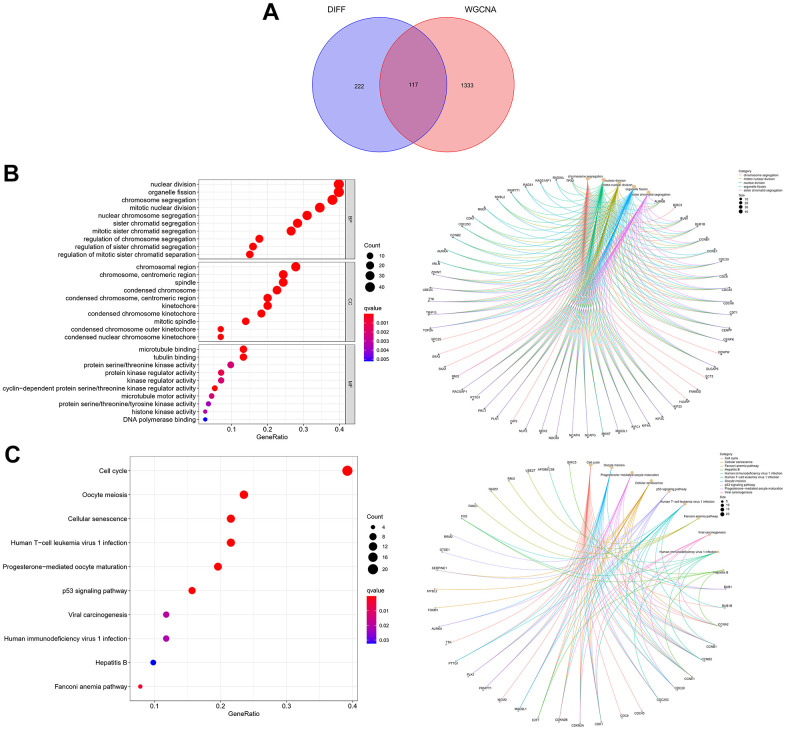
**Expression of the 117 cellular senescence-related DEGs and their functions.** (**A**) The Venn diagram shows an overlap of the differential analysis and WGCNA results. (**B**) GO function analysis. (**C**) KEGG pathways analysis.

The “sva” tool in R removed the sample batch effect (371 tumors and 160 normal tissues) from the TCGA-LIHC and GTEx datasets. After expression normalization, PCA showed no batch effect (Figure 1A, 1B). In the merged dataset, 309 up-regulated and 31 down-regulated cellular senescence-related genes were found (Figure 1C and Supplementary Table 6). WGCNA reveals gene expression patterns and major gene modules from numerous samples to investigate the essential module correlated to the liver’s pathological state. The combined dataset was filtered and utilized for sample hierarchical clustering using the average linkage approach to evaluate outlier samples (Figure 2A). According to the WGCNA approach, an ideal value for the merged dataset’s soft power was found to be β= 8 (Figure 2B). 10 modules were recognized in the combined dataset. After, calculations were made to determine the relationships between the module and the pathological state. The Pearson correlation heatmap suggested that the turquoise and brown modules significantly correlated with liver pathology and were selected for future study (Figure 2C). Scatter plots showed gene significance with module membership (Figure 2D). In Figure 3A, 117 genes from differential analysis and WGCNA overlapped. Those overlapped genes were considered cellular senescence-related DEGs. Moreover, the biological process of cellular senescence-related DEGs mainly includes nuclear division, organelle fission, and chromosome segregation. Meanwhile, the molecular functions were chromosomal region, spindle, and condensed chromosome. Cellular components mainly comprised microtubule binding, tubulin binding, and protein kinase regulator activity (Figure 3B). Cell cycle, oocyte meiosis, cellular senescence, and the p53 signaling pathway were considerably enriched, according to an assessment performed by KEGG pathways (Figure 3C).

### Construction of machine learning based prognostic model

**Figure 4:**
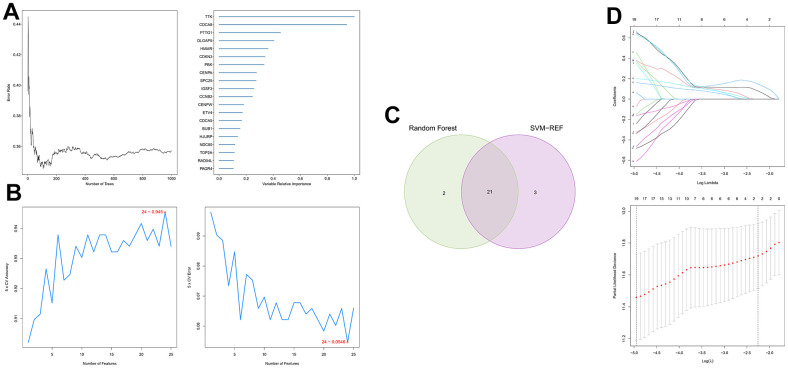
**Generation of a cellular senescence score model using machine learning.** (**A**) Error plot for RF models. (**B**) Screening of candidate genes by SVM models. (**C**) Twenty-one marker genes in RF and SVM models were intersected in the Venn diagram. (**D**) LASSO regression analysis.

**Figure 5:**
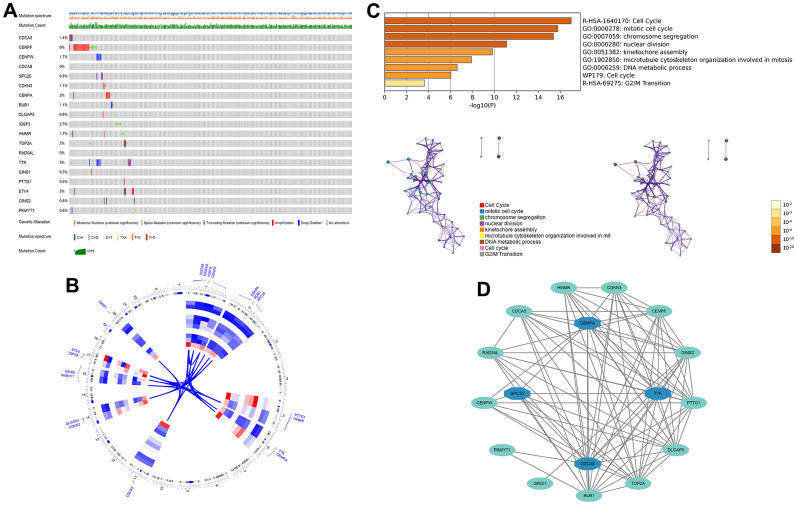
**The genetic alterations and biological functions analysis of the nineteen prognostic genes in HCC.** (**A**) The genetic alteration profiles of the 19 genes in the TCGA-LIHC dataset using the cBioPortal database. (**B**) Circular visualization of chromosomal positions of 19 genes. (**C**) Biological functions analysis of 19 genes in Metascape database and nodes with the same color belong to the same term. (**D**) Hub genes in the protein-protein interaction network.

First, 26 DEGs related to cellular senescence were identified as associated with HCC patient prognosis by univariate and multivariate Cox analyses (Supplementary Table 7). According to above steps, RF with SVM was used to screen 26 candidate genes from differentially expressed genes related to cellular senescence (Figure 4A, 4B). After intersecting the marker genes generated from the RF and SVM models, 21 marker genes were found worthy of further investigation (Figure 4C). Next, we penalized 21 marker genes by LASSO cox regression (Figure 4D). As a result, 19 genes, including CDCA5, CENPF, CENPW, CDCA8, SPC25, CDKN3, CENPA, BUB1, DLGAP5, IGSF3, HMMR, TOP2A, RAD54L, TTK, GINS1, PTTG1, ETV4, GINS2, and PKMYT1 were found to be independent prognostic biomarkers in HCC (Supplementary Table 8 and Supplementary Figure 3). The nineteen genes expression profile generated the senescence score = (-0.24208*CDCA5 exp) + (-0.32203*CENPF exp) + (-0.15081*CENPW exp) + (0.66330*CDCA8 exp) + (0.35072*SPC25 exp) + (-0.46527*CDKN3 exp) + (0.64921*CENPA exp) + (-0.07426*BUB1 exp) + (0.37272*DLGAP5 exp) + (0.16174*IGSF3 exp) + (0.63189*HMMR exp) + (-0.61190*TOP2A exp) + (-0.33728*RAD54L exp) + (0.37964*TTK exp) + (0.45727*GINS1 exp) + (0.06315*PTTG1 exp) + (0.20073*ETV4 exp) + (-0.16337*GINS2 exp) + (-0.48263*PKMYT1 exp). The genetic alterations that occurred most frequently in nineteen genes were amplification and deep deletion (Figure 5A). Chromosome mapping of the consensus genes revealed genome-wide distribution, with chromosomes 1, 2, 5, 6, 14, 16, and 17 containing the most significant number of dysregulated cellular senescence-related genes in HCC. In contrast, no X and Y chromosome gene was affected (Figure 5B). These 19 genes were further subjected to a Metascape and MCODE analysis to determine biological significance in the pathogenesis of HCC. According to Metascape analysis, those genes were predominantly involved in the cell cycle, chromosome segregation, nuclear division, and other critical pathways, most of which were related to DNA metabolism (Figure 5C). The MCODE analysis determined that CENPA, SPC25, CDCA8, and TTK were hub genes among nineteen cellular senescence genes (Figure 5D). Moreover, we tested the diagnostic efficacy of those biomarkers in GSE121248 and GSE45267 datasets. The results revealed that they exhibit considerable expression discrepancies (Supplementary Figure 4). Two biomarkers showed promising diagnostic values in training set: CENPW (AUC = 0.971), TOP2A (AUC = 0.958) (Supplementary Figure 5A). Validation in the external set (GSE121248 and GSE45267) also confirmed these findings. The diagnostic accuracy for detection of HCC in GSE45267 dataset: CENPW (AUC = 0.980), TOP2A (AUC = 0.982) (Supplementary Figure 5B). The validation datasets GSE121248 also corroborated the following findings: CENPW (AUC = 0.942), TOP2A (AUC = 0.947) (Supplementary Figure 5C).

### Evaluating the predictive capacity for cellular senescence-related gene prognostic signature

**Figure 6:**
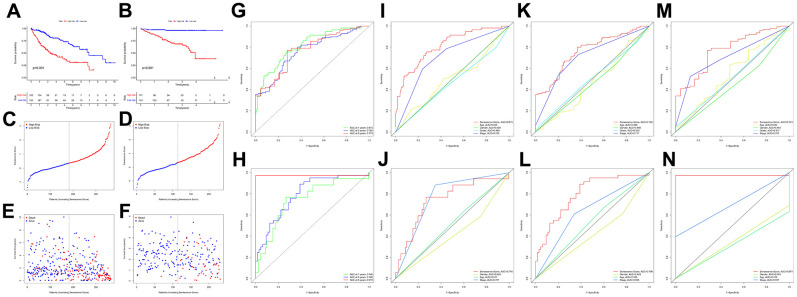
**Survival analysis of the senescence score model.** KM survival analysis for the senescence score model in TCGA-LIHC (**A**) and HCCDB18 (**B**) dataset. Survival status plots of HCC patients in TCGA-LIHC (**C**) and HCCDB18 (**D**) datasets. Senescence score distribution plots of HCC patients in TCGA-LIHC (**E**) and HCCDB18 (**F**) datasets. Time-dependent ROC analysis at 1-,3-, and 5-year follow-up in TCGA-LIHC (**G**) and HCCDB18 (**H**) datasets. Clinical characteristics and senescence score model ROC analysis at 1-year follow-up in TCGA-LIHC (**I**) and HCCDB18 (**J**) datasets. Clinical characteristics and senescence score model ROC analysis at 3-year follow-up in TCGA-LIHC (**K**) and HCCDB18 (**L**) datasets. Clinical characteristics and senescence score model ROC analysis at 5-year follow-up in TCGA-LIHC (**M**) and HCCDB18 (**N**) datasets.

The results of the KM analysis showed that patients with a high risk had worse clinical outcomes than those with low risk (Figure 6A, 6B). At the same time, in the TCGA-LIHC cohort, the area under the receiver operating characteristic curve (AUC) for the prognostic prediction power of the senescence score model were 0.815 (1 year), 0.762 (3 years), and 0.773 (5 years) (Figure 6G). Besides, in the validation cohort, our model produced the following results for AUC: 0.746 (1 year), 0.796 (3 years), and 0.974 (5 years) (Figure 6H). Furthermore, the senescence score model outperformed pathological characteristics in predicting HCC patients’ prognoses in the TCGA-LIHC (Figure 6I, 6K, 6M) and HCCDB18 (Figure 6J, 6I, 6L, 6N) cohorts. Moreover, the hazard survival status plots between two subgroups in both cohorts demonstrated the increase of risk value of the novel prediction model with decreased patients’ overall survival rate (Figure 6C–6F). Since many research teams have also proposed multi-gene signatures for HCC prognosis, their signatures have apparent limitations. Their performance was evaluated parallel to our nineteen genes’ signature using time-dependent ROC curves and C-indexes. Our nineteen genes’ signature had the highest predictive efficiency when all the prediction signatures were compared (Table 1).

**Table 1:** Comparison of recent gene signatures built for predicting overall survival in HCC patients.

**Study**	**Our study**	**PMID: 32198063**	**PMID: 35123387**	**PMID: 35123420**	**PMID: 35705729**	**PMID: 35699863**	**PMID: 35402624**	**PMID: 33758763**	**PMID: 35535359**
Statistical methods	Univariate Cox Multivariate Cox SVM-REF Random Forest LASSO Cox	PPI network MCODE Multivariate Cox	Univariate Cox LASSO Cox	Univariate Cox LASSO Cox Multivariate Cox	Univariate Cox LASSO Cox Multivariate Cox	Univariate Cox LASSO Cox	Univariate Cox LASSO Cox	Univariate Cox LASSO Cox Multivariate Cox	Univariate Cox LASSO Cox
Training cohorts									
1-year AUC	0.815	0.710	0.770	0.851	0.683	0.767	0.734	0.805	0.790
3-year AUC	0.762	0.740	0.713	0.727	0.559	0.680	0.692	0.803	0.770
5-year AUC	0.775	0.640	0.693	0.691	—	—	0.663	—	0.770
Validation cohorts									
1-year AUC	0.746	0.640	0.641	0.705	0.534	0.677	—	0.721	0.780
3-year AUC	0.796	0.590	0.663	0.717	0.635	0.689	—	0.693	0.740
5-year AUC	0.974	0.650	0.681	0.684	—	—	—	0.737	0.780
C-index	0.741	0.692	0.658	0.706	0.634	0.646	0.675	0.671	0.714

### Independent prognostic value evaluation of cellular senescence-related genes prognostic signature

**Figure 7:**
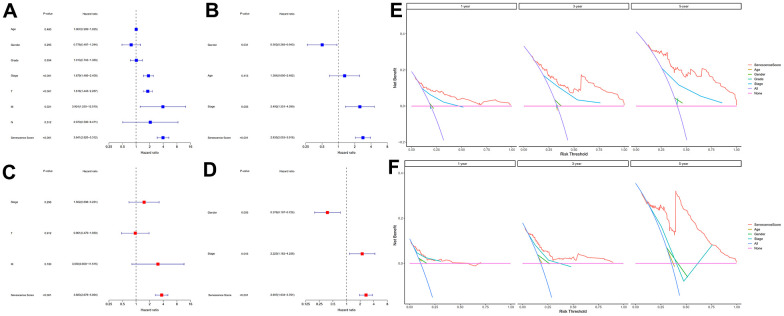
**Evaluation of the senescence score model's prognostic accuracy.** Univariate independent Cox analyses in TCGA-LIHC (**A**) and HCCDB18 (**B**) datasets. Multivariate independent Cox analyses in TCGA-LIHC (**C**) and HCCDB18 (**D**) datasets. The decision curve analyses of the senescence score model in TCGA-LIHC (**E**) and HCCDB18 (**F**) datasets.

**Figure 8:**
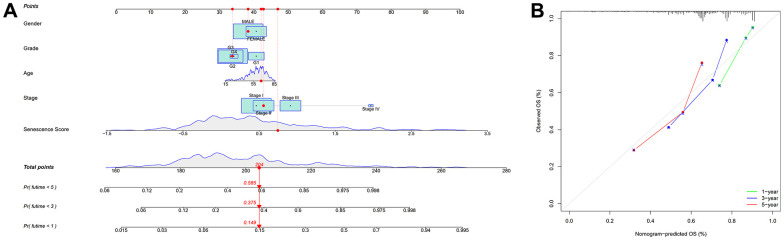
**Nomogram based on senescence score model.** (**A**) Nomogram. (**B**) The nomogram’s calibration curves.

We used univariate and multivariate cox analysis to determine if the cellular senescence-related genes signature was an independent survival predictor. Patients’ overall survival was substantially correlated with their senescence score in univariate cox analysis (TCGA cohort: HR = 3.941, 95% CI = 2.3925-5.312, P < 0.001; HCCDB18 cohort: HR = 2.835, 95% CI = 2.053-3.916, P < 0.001) (Figure 7A, 7B). Multivariate Cox analysis determined the cellular senescence score model to be an independent patient prognostic factor (TCGA cohort: HR = 3.683, 95% CI = 2.678-5.064, P < 0.001; HCCDB18 cohort: HR = 2.697, 95% CI = 1.934-3.761, P < 0.001) (Figure 7C, 7D). Heatmap showed the senescence score model’s nineteen genes’ clinical characteristics (Supplementary Figure 6). Additionally, the DCA evaluation revealed that the senescence score model performed better than other clinicopathological characteristics in predicting patients’ overall survival (Figure 7E, 7F). The accuracy of the nomogram in predicting patient outcomes was proven by the fact that the calibration curves had an optimal degree of fitting to the observed data (Figure 8A, 8B). As a result, this innovative senescence score model can effectively forecast HCC patients’ prognoses.

### Gene set enrichment analysis

**Figure 9:**
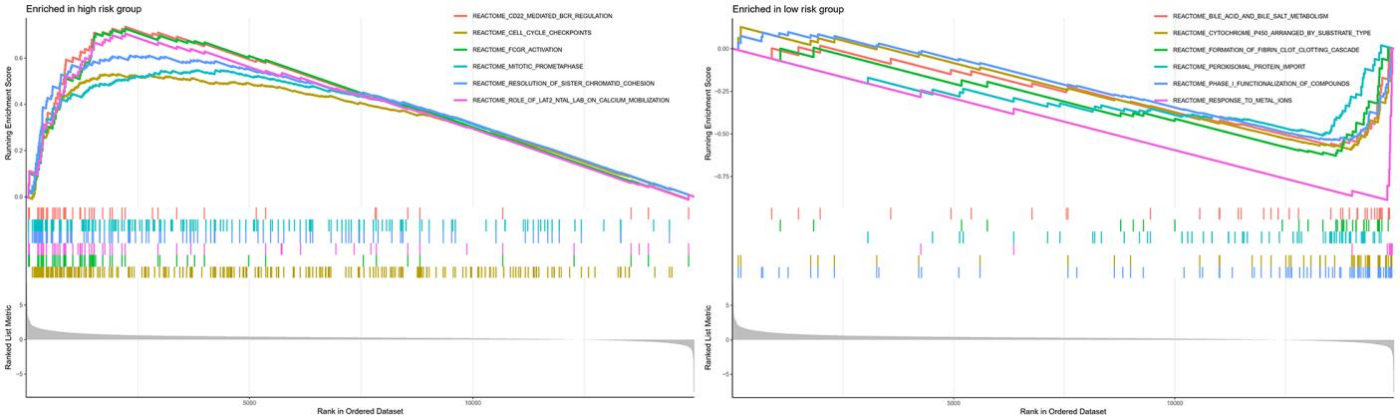
GSEA analyses between different cellular senescence-related genes signature risk groups.

GSEA analysis indicated that CD22-mediated BCR modulation, cell cycle checkpoints, FCGR activation, and mitotic prometa centrally involve in regulating neoplasm development and immune response in high-risk individuals (Figure 9). Meanwhile, metabolic-related biological processes and pathways in the individuals of low-risk group were mainly cytochrome p450 arranged by substrate type, peroxisomal protein import, and response to metal ions. This implied that the worse prognosis of high-risk individuals could be driven by the further activation of immune signaling suppression in tumor cells and the dysregulation of oxidative metabolism.

### Analysis of immune response

**Figure 10:**
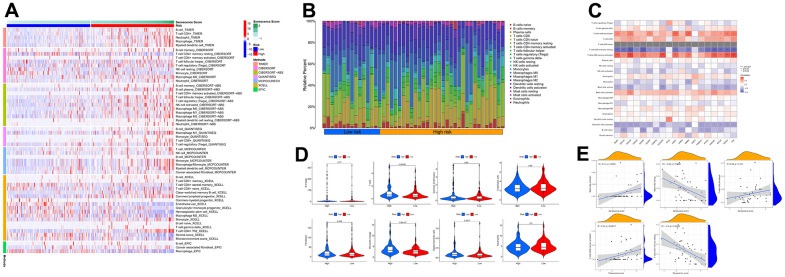
**The immune response of the cellular senescence-related genes signature.** (**A**) The immune infiltration status of the high-risk and low-risk groups. (**B**) The ratio of 22 immune cells components of the two risk groups. (**C**) Heat map depicting the relationship between the 19 genes associated with cellular senescence and immune cells. (**D**) Comparison of immune infiltration calculated using “MCPcounter” between two risk groups. (**E**) Relevance between senescence score and immune cells response.

**Figure 11:**
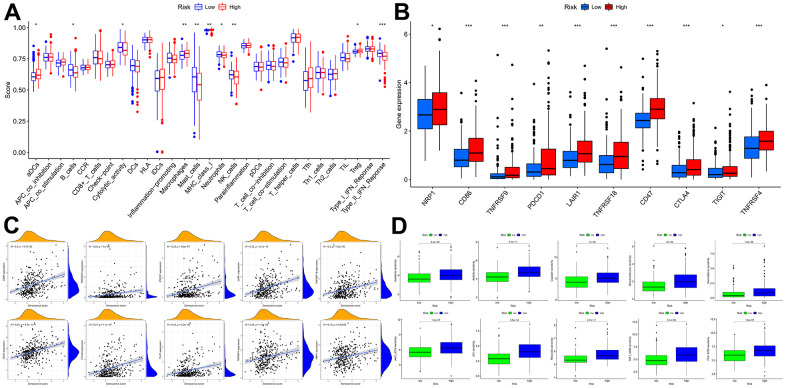
**Immune function and immunotherapy comparisons between the two risk groups.** (**A**) Immune function analysis. (**B**) Immune checkpoint analysis between risk groups. (**C**) Relevance between senescence score and immune checkpoints expression. (**D**) Comparison sensitivity of targeted therapeutic drugs of individuals in different risk groups.

Based on multiple immune algorithms, the heatmap and bubble plot represented that the immune cells’ response expression was upregulated in patients with a high risk of HCC (Figure 10A and Supplementary Table 9). To explore the two risk groups’ immune response, we evaluated the immune cells’ relative percentage of each sample among immune cells in CIBERSORT. The relative percentage of the immune cells differed within and between groups (Figure 10B). According to our analysis of the relationship between nineteen cellular senescence-related genes signature and immune cells infiltration, CDCA5, CENPF, CENPW, CDCA8, SPC25, CDKN3, CENPA, BUB1, DLGAP5, HMMR, TOP2A, RAD54L, TTK, GINS1, PTTG1, GINS2, and PKMYT1 were positively associated with T cells CD4 memory activation, negatively associated with T cells’ CD4 memory resting. The resting state of dendritic cells was found to have a favorable association with ETV4. GINS1 and HMMR were positively correlated with T cells follicular helper (Figure 10C). Assessment of MCPcounter showed that high-risk patients had more T cells, fibroblasts, monocytic lineage, and myeloid dendritic cells infiltration than low-risk patients. (Figure 10D). Pearson’s correlation analysis results indicated that our senescence score significantly correlated with the expression level of immune cells. Mast cells activated, T cells follicular helper, and T cells CD4 memory activated linked positively with senescence scores, while mast cells resting and T cells CD4 memory resting correlated negatively. Single-sample gene set enrichment analysis showed significant immune function differences between two risk subgroups. (Figure 11A). The immune functions most significantly upregulated in high-risk were Treg, Macrophages, aDCs, and MHC class-I. In contrast, high-risk group down-regulated B cells, mast cells, neutrophils, cytolytic activity, type II INF response, and NK cells, implying that the cellular senescence reduced liver cancer cell susceptibility to NK cell cytotoxicity and inhibition of IFN production and released leads to hepatocellular carcinomatous growth.

In light of the significance of immunotherapy based on checkpoint inhibitors in treating HCC, the amount of immune checkpoint expression was investigated in two groups (Figure 11B, 11C). In comparison, immunological checkpoints were more actively expressed in high-risk individuals, thus suggesting a better response to immunotherapy. Moreover, “oncoPredict” tool assessed high- and low-risk patients’ targeted therapy responsiveness. The results showed that high-risk and low-risk individuals had considerably different estimated IC50s for the ten targeted therapy medications (Figure 11D). It suggested that high-risk HCC patients were more susceptible to Sorafenib, Axitinib, Dihydrorotenone, JQ1, and TAF1, further refining the medication range.

### Pseudo-time and trajectory analysis revealed dynamic change of hub genes in cellular senescence-related prognostic signature

**Figure 12:**
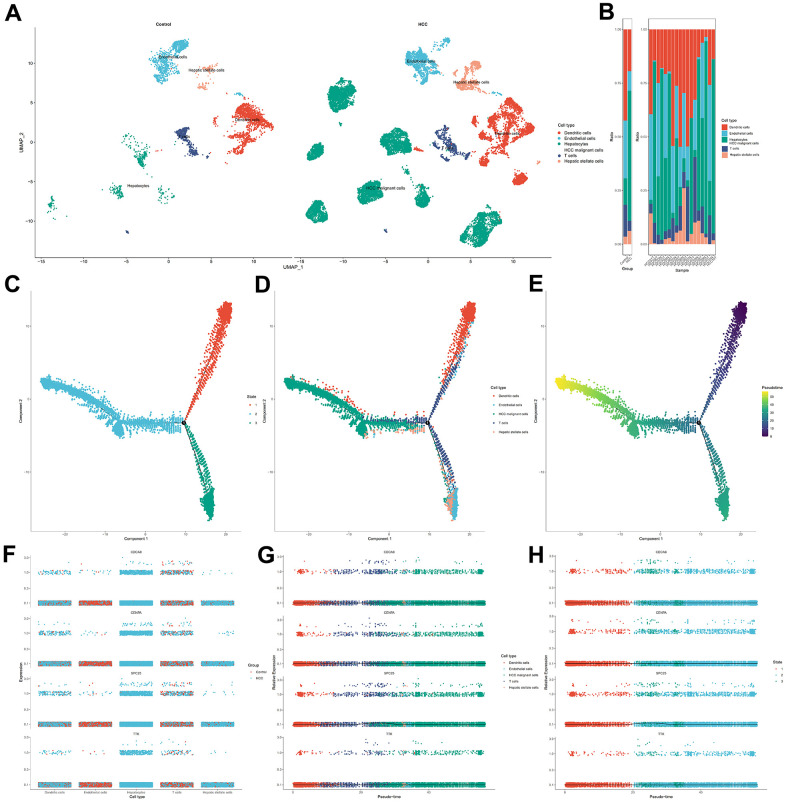
**Single cell analysis for the four hub genes in cellular senescence-related prognostic signature.** (**A**) Visualization of cell types in non-tumor liver specimens and HCC specimens. (**B**) The frequency of cells in two groups. (**C**) Cells in HCC were colored based on state. (**D**) Distribution of cells in HCC in pseudo-time trajectory. (**E**) Cells were colored on the basis of pseudo-time. (**F**) The dithering plot shows the expressions of the four hub genes in five cell types between control and HCC samples. (**G**) Pseudo-time analysis for expression kinetics of four hub genes in cell types trajectory. (**H**) Pseudo-time analysis for expression kinetics of four hub genes in different states trajectory of cells.

The cells of HCC samples and non-tumor liver samples (control group) were classified into 15 clusters via UMAP algorithm (Supplementary Figure 7). Then, Dendritic cells, endothelial cells, T cells, hepatic stellate cells, hepatocytes, and HCC malignant cells were the cell types assigned to the 15 clusters (Figure 12A). The frequency of cell types between two specimens shows that dendritic and T cells were predominant in control group cells (Figure 12B). Next, the cells in the HCC group were assigned to three states with one main path by pseudo-time and trajectory analysis (Figure 12C, 12D and Supplementary Figure 8). State one mainly contained dendritic cells. State two mainly contained HCC malignant cells. State three mainly contained endothelial cells and hepatic stellate cells. T cells were positioned in three timeline trajectories. Pseudo-time flows for distributions of cell states are displayed in Figure 12E. The color gradient indicates the direction of pseudo-time flow. The root of the cell differentiation trajectory is at state one and then partitioned into state two and state three at the intersection point. According to the results from the above steps, CDCA8, CENPA, SPC25, and TTK were hub genes in cellular senescence genes signature. Therefore, we analyzed them further in single cells. CENPA and TTK were overexpressed in the dendritic and T cells of HCC samples. Only CDCA8 of the four hub genes is expressed higher in normal liver endothelial cells than in HCC samples. The expression profiles of CDCA8, CENPA, SPC25, and TTK in malignant hepatocytes were higher than in normal hepatocytes. HCC hepatic stellate cells expressed all four hub genes substantially higher than controls (Figure 12F). The dynamic expression patterns of the four hub genes along the time trajectory of HCC progression were further analyzed. Pseudo-time series analysis showed that the expression values of CDCA8, CENPA, SPC25, and TTK were relatively high in malignant cells and immune cells at the late stage of differentiation, indicating that their expression direction was consistent with the progression of HCC toward poor prognosis (Figure 12G, 12H and Supplementary Figure 9).

## DISCUSSION

Cellular senescence is a reasonably permanent state in which cells irreversibly decouple from the cell cycle and lose proliferative potential as a result of continuous stress-induced damage [29]. Many changes occur in the liver as a result of aging and various stressors, such as oxidative stress or oncogene activation, including a decrease in the size and a total number of normal hepatocytes, a decrease in regenerative and metabolic capacity, and an increase in the proportion of polyploid and multinucleated hepatocytes [30, 31]. Clinical studies have shown that hepatocyte senescence occurs *in vivo* in patients with HCC [32]. Hepatocyte senescence slows the proliferation of injured hepatocytes, ensuring a stable halt in proliferation and division and causing changes in the microenvironment and homeostasis. Early in cancer, senescence-associated signaling pathways undergo regulatory dysfunction, rendering damaged cells unable to senesce normally, and the cell cycle becomes uncontrolled [33]. It follows that cellular senescence may be a possible anticancer mechanism.

The primary object of this research was to explore the function of cellular senescence in determining the prognosis for HCC and in developing treatments for the disease. Our analysis first uncovered 117 cellular senescence-related DEGs in HCC and non-neoplastic tissues. These DEGs were found to be involved in the cell cycle, nuclear division, organelle fission, chromosomal segregation, mitotic nuclear division, and the p53 signaling pathway. These results are compatible with those reported in previous experiments. Cell senescence is regulated by p53 / p21 and p16INK4a signaling pathways [34]. As a natural barrier to tumor suppression, p53 tumor suppressor limits malignant transformation by triggering cell-autonomous programs such as cell cycle arrest or apoptosis [35]. The senescence state triggered by p53 leads to a significant increase in the secretion of factors that promote M1 polarization, enabling IL1 β Expression and an increase in the propensity for cell killing and phagocytosis, inhibiting the secretion of M2 polarization-related factors [36].

To further investigate cellular senescence-related predictive therapeutic biomarkers and identify novel effective therapeutic targets in HCC, we created a nineteen cellular senescence-related genes model, senescence score using multiple machine learning algorithms. The cellular senescence score model identified high-risk and low-risk HCC patients with different survival rates. Survival analyses indicated high-risk groups with worse HCC prognoses. Correspondingly, the senescence scoring system was practiced well in the external validation dataset and outperformed existed prognosis classifier in HCC. Finally, hybrid nomogram incorporating a senescence score model applied in predicting HCC prognosis was robust in evaluation. Acquired nineteen cellular senescence-related prognostic biomarkers were shown to play corresponding roles in the cell cycle via the “metascape” annotation tool analysis. Among nineteen cellular senescence-related genes signature, CENPA, SPC25, CDCA8, and TTK were hub genes. As a histone H3 variant of centromeric nucleosomes, CENPA must ensure that kinetochores are used for correct chromosome separation and assembly [37]. The aberrant expression or functional defect of CENPA leads to the interruption of genome integrity and abnormal cell division, thus inducing the emergence of cancer [38]. Previous literature [39] showed that CENPA was abnormally overexpressed in HCC tissues [40], consistent with our findings. Basic experimental researches have demonstrated a reduction in CENPA levels can stifle the growth of HepG2 cells, ending the cell cycle in the G1 phase and leading to apoptosis. In contrast, CENPA overexpression promoted the growth of HCC cells and reduced apoptosis. The present study revealed that a correlation between the upregulation of SPC25 expression and increased cell proliferation and poor prognosis in HCC patients. From a molecular aspect, SPC25 is one of four proteins that make up the nuclear division cycle 80 (NDC 80) complex, playing a crucial role in the assembly of kinetochore microtubule-binding domain and mediates the alignment of chromosomes with the metaphase plate [41]. Dysfunction of the NDC 80 complex due to various factors can lead to abnormal chromosome segregation, affecting cell division and ultimately resulting in abnormal proliferation [42]. As a critical component of the NDC 80 complex, the induction of disorganized cell mitosis by SPC25 overexpression leads to enhanced proliferative capacity and deepening of malignancy in tumor cells and further worsens the prognosis of patients with tumors [43]. CDCA8 encodes Borealin/Desra B protein, an essential component of chromosome passenger complex [44]. It is crucial in locating chromosome passenger complex to the centromere, correcting kinetochore binding errors, and stabilizing bipolar spindles [45]. Studies have reported that the transcriptional activity of CDCA8 was increased in embryos, embryonic stem cells, and cancer cells, and its expression was very weak or not expressed in normal tissues [46]. The experiment proves that CDCA8 regulates HCC cells’ proliferation via activating cell cycle, and Huh7 cells that knock out CDCA8 are blocked in G0/G1 phase, inhibiting the proliferation of HCC cells [47]. Meanwhile, the mitotic checkpoint and faulty chromosomal linkages depend on TTK’s dual serine/threonine and tyrosine protein kinase. As a potential oncogene, its elevated expression level leads to centrosome amplification, hyperactivation of the mitotic spindle checkpoint, and chromosomal instability, resulting in tumorigenesis [48]. *In vitro* and *in vivo* functional experimental assays showed that TTK overexpression promoted HCC cell proliferation and formed resistance to sorafenib. Either depletion or activity inhibition of TTK significantly inhibited the viability of HCC cells [49, 50]. Other fifteen genes were also identified to contribute to HCC carcinogenesis and progression [51–55].

In neoplastic process, cellular senescence intersects at many levels with the immune responses [56, 57]. GSEA analysis revealed that the high-risk group enriched immunological and tumor-related pathways, indicating two distinct effects of cellular senescence on HCC cells biogenesis and death. The results of multi-immune algorithms indicated that the cellular senescence model was closely associated with immune cell infiltration. And HCC patients with worse clinical outcome had higher immune infiltration than low-risk patients. Tumorigenesis occurs via evading autoimmune-mediated elimination triggered by senescent cells’ senescence-associated secretory phenotype [58]. CD4^+^ T cells are resistant to age-related phenotypic and functional changes, and a gradual increase in the percentage of senescent-like CD4^+^ T lymphocytes is generally seen when an individual ages [59]. Functional alterations in subsets of human tumor-induced senescent CD4^+^ T cells, which inhibit the proliferation of responder T cells through the cell-to-cell contact, are tumor-promoting mechanisms [60]. Tumor cells induce senescence T cells to secrete pro-inflammatory cytokines that induce premature senescence of surrounding cells through a paracrine mechanism, allowing senescent T cells to increase in the tumor microenvironment [61]. High levels of senescent T cells predict poor prognosis in tumors. A previous study [62] showed that senescent melanoma cells could activate dendritic cells through direct cellular contact, allowing them to acquire and present antigens more efficiently. Compared with a non-senescent cell environment, dendritic cells in a senescent cell environment are better able to activate OT-I CD8^+^ T cells, resulting in strong anti-tumor protection. Furthermore, an immune function analysis in this study suggested significant attenuation of B cells, mast cells, neutrophils, cytolytic activity, type II INF response, and NK cells at high risk of HCC, indicating that suppression of antitumor immunity reaction results in a poor prognosis. It follows that boosting the innate antitumor immune responses is crucial for halting the HCC progression and devising effective treatments. Studies have shown that artificially induced senescent cells secrete pro-inflammatory senescence-associated secretory phenotype factors, which further recruit various immune cells, infiltrate the periphery of diseased tissues, activate immune surveillance, rapidly recognize and clear senescent cells, and block tumorigenesis [63–65]. The effect of senescence on the efficacy of immune checkpoint inhibitors remains insufficiently evaluated in current preclinical studies. In this study, PDCD1, CTLA4, TIGIT, LAIR1, CD47, TNFRSF4, TNFRSF9, and TNFRSF18 were upregulated in the high-risk group, reflecting HCC’s immunosuppressive microenvironment. The levels of expression of immunological checkpoints growing in synchrony with senescence scores might explain why patients who respond to immune checkpoint blockade show stable growth arrest of tumors rather than complete tumor regression. Because the senescence response is frequently inactive in cancer cells, activating it is an essential and potentially fruitful technique for treating tumors [66]. Oncogene-induced senescence maintains tumor cells in a non-invasive, pre-malignant stage, restricting future cell development, whereas cells that do not generate senescence responses advance to a malignant state [33, 67, 68]. The increasing prevalence of HCC and the difficulties in treating it due to a lack of approved drugs highlight the critical need for novel pharmacological strategies and systemic therapy [69]. Inducing senescence is a unique way to treat HCC, especially with medications that kill senescent cancer cells [70]. Therefore, based on the senescence score, we investigated the treatment sensitivity of immunotherapy drugs in a population of patients at different risks for HCC. Our research discovered that the individuals with worse prognoses were more susceptible to ten drugs, including Sorafenib, Axitinib, Cisplatin, Dihydrorotenone, Gemcitabine, JAK1_8709, JQ1, Ribociclib, TAF1_5496, PLX-4720. One study found [71] that Sorafenib therapy of doxorubicin-induced senescent cancer cells altered the sensitivity to apoptosis and decreased the number of SA-β-gal positive cells, indicating the possible senolytic effect of sorafenib in these cells. Axitinib has also been demonstrated to reduce cell proliferation and delay tumor growth by inducing cell cycle arrest, senescence, apoptosis, and antiangiogenesis in the G2/M phase [72]. Meanwhile, it has been shown that Ribociclib interferes with cell cycle progression, induces cellular senescence, and promotes cancer cell destruction through cytotoxic T-cell-mediated effects [73]. This evidence may also contribute to guiding targeted therapies for HCC.

Although we determined and validated the utility of a profile of a cellular senescence score prognostic model for HCC, our bioinformatics research has several limitations. Although cellular senescence-associated differential genes in HCC were examined at the transcriptional level, they have not been validated at the protein level. Multi-omics studies, such as proteome and metabolomics, may provide additional insight into its mechanism. Since the data samples from public databases were retrospective, the inherent case selection bias that may have influenced the outcomes. The mechanism of prognostic-associated cellular senescence genes in HCC and immune activity needed further large samples of experimental exploration.

## CONCLUSIONS

In conclusion, this study constructed an advanced machine-learning based cellular senescence-related gene signature, a reliable prognosis predicting approach for HCC patients, and sheds light on future targeted therapeutics.

## Supplementary Material

Supplementary Figures

Supplementary Table 1

Supplementary Table 2

Supplementary Table 3

Supplementary Table 4

Supplementary Table 5

Supplementary Table 6

Supplementary Table 7

Supplementary Table 8

Supplementary Table 9
