# Supplementary material for: Cellular senescence-related gene signature as a valuable predictor of prognosis in hepatocellular carcinoma
Source: Aging (Albany NY). 2023 Apr 13;15(8):3064–93. doi: 10.18632/aging.204658 (PMC10188344; doi:10.18632/aging.204658)
Supplement: Supplementary Figures [file aging-15-204658-s001.pdf]

SUPPLEMENTARY FIGURES

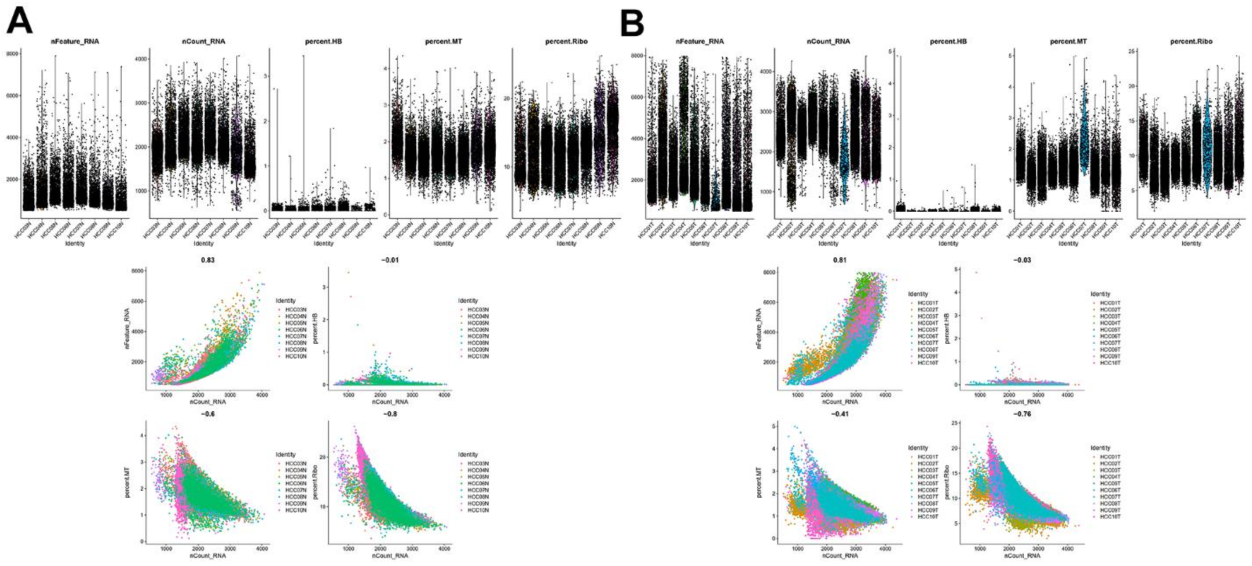

Supplementary Figure 1. Quality control of the single cell data. (**A**) The number of genes and relative hemoglobin, mitochondrial, and ribosomal transcript abundance from non-tumor liver cells. (**B**) The number of genes and relative hemoglobin, mitochondrial, and ribosomal transcript abundance from HCC cells.

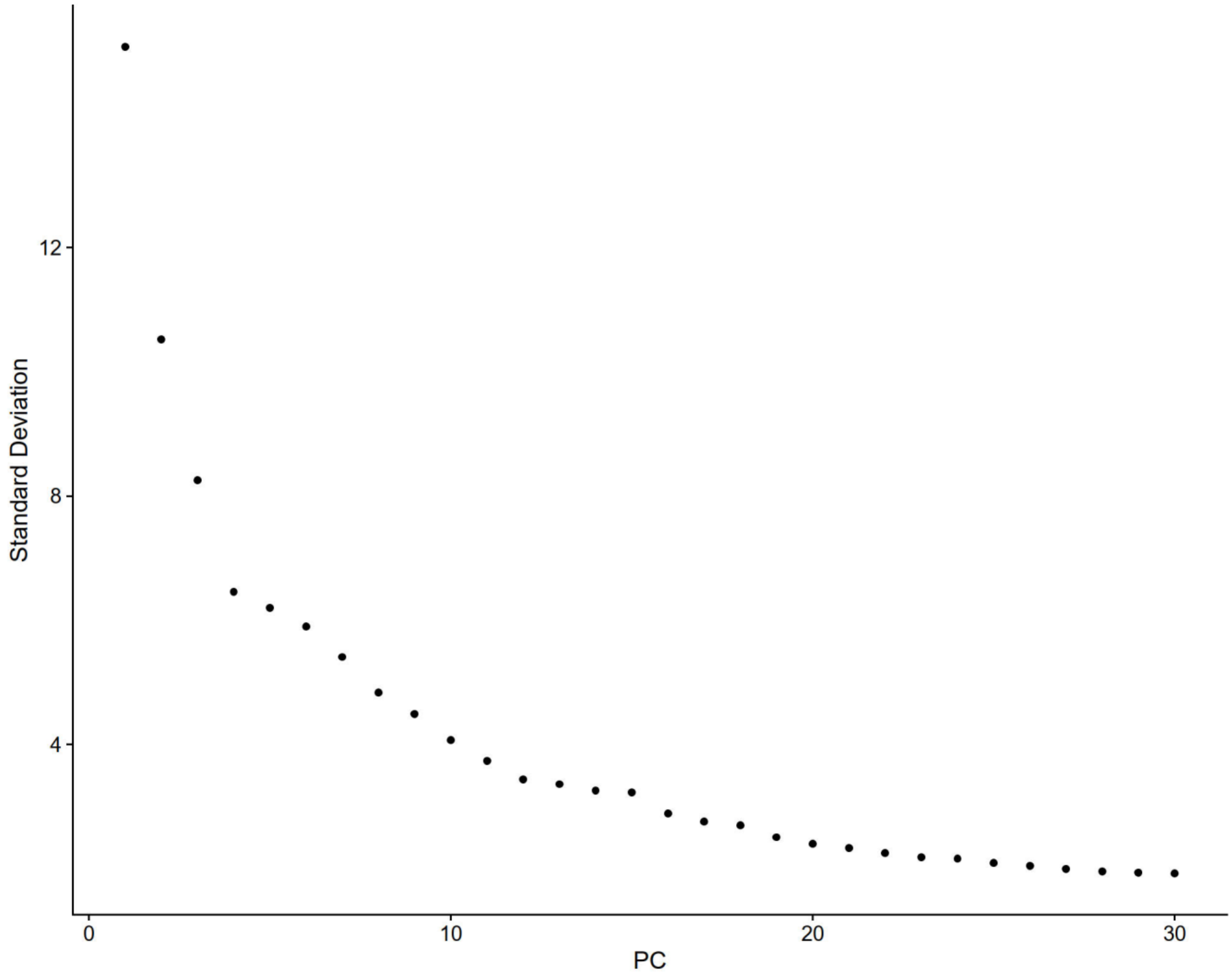

Supplementary Figure 2. The elbow plot of PCA analysis.

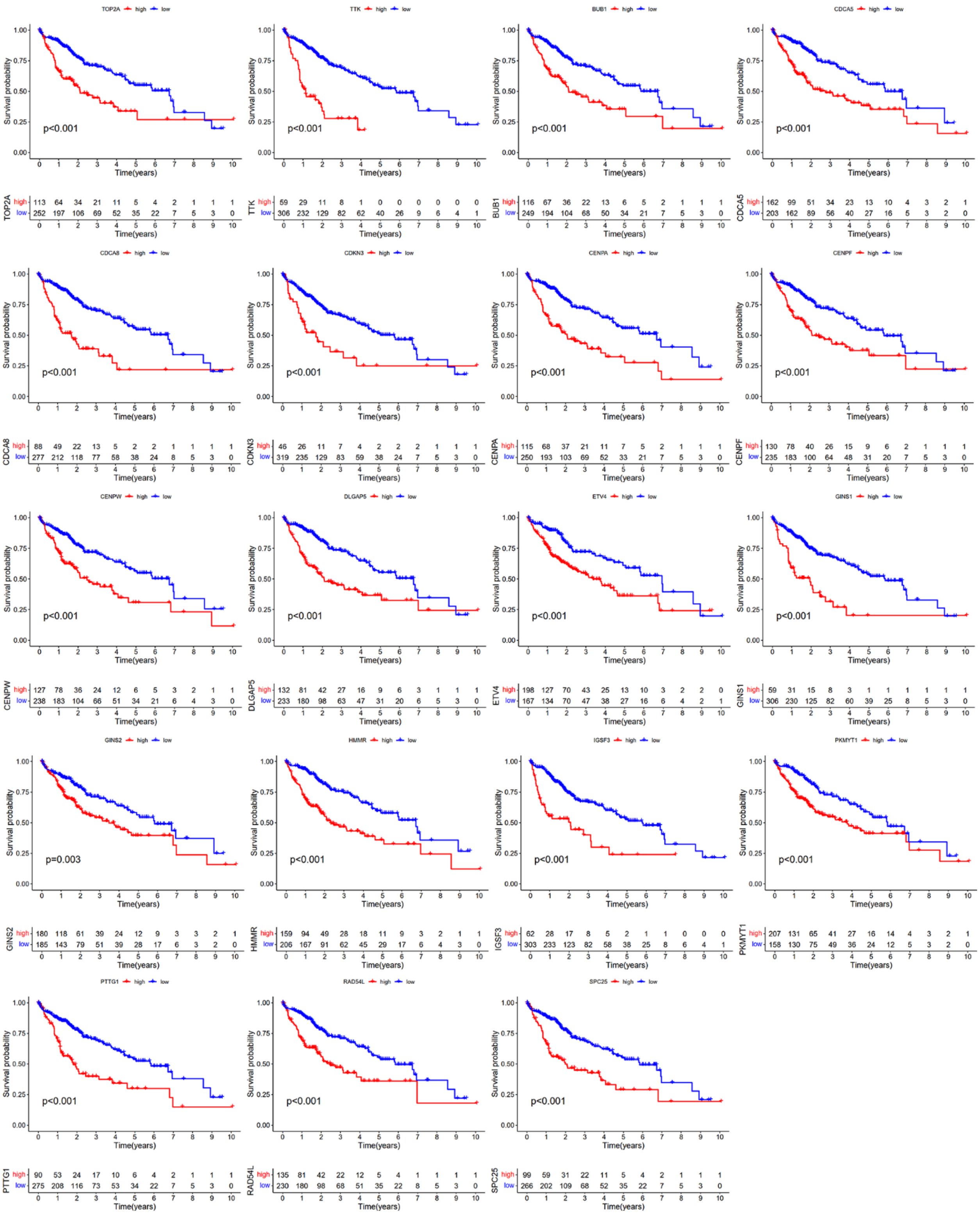

Supplementary Figure 3. KM analysis of 19 cellular senescence-related genes signature.

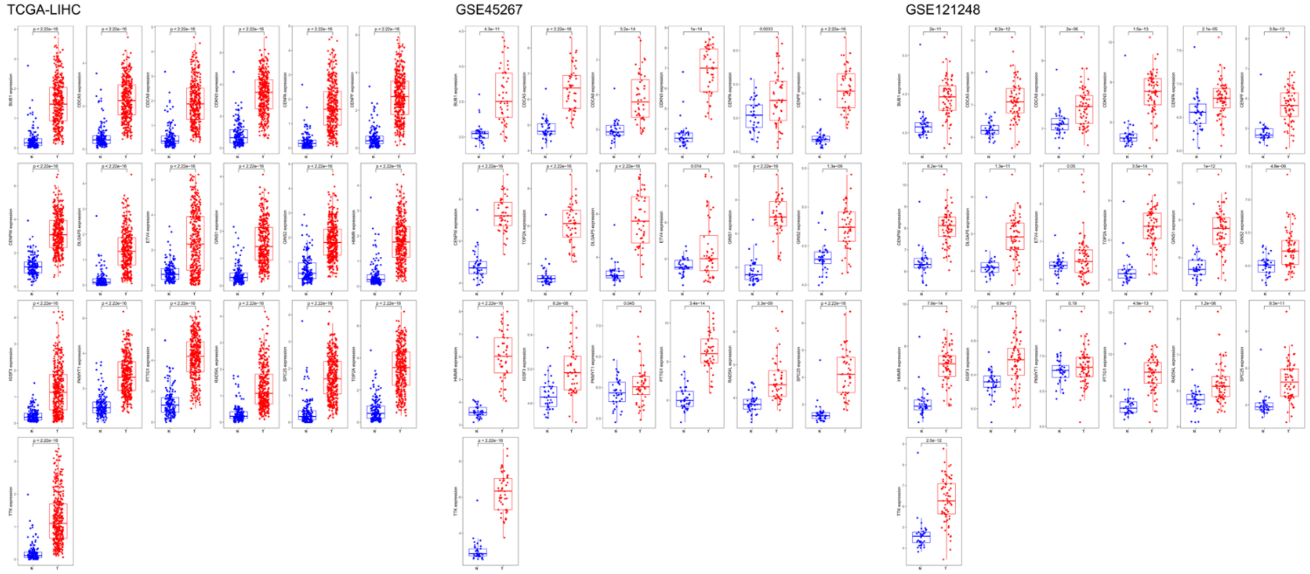

Supplementary Figure 4. The 19 cellular senescence-related genes' signature expression in training and validation cohorts.

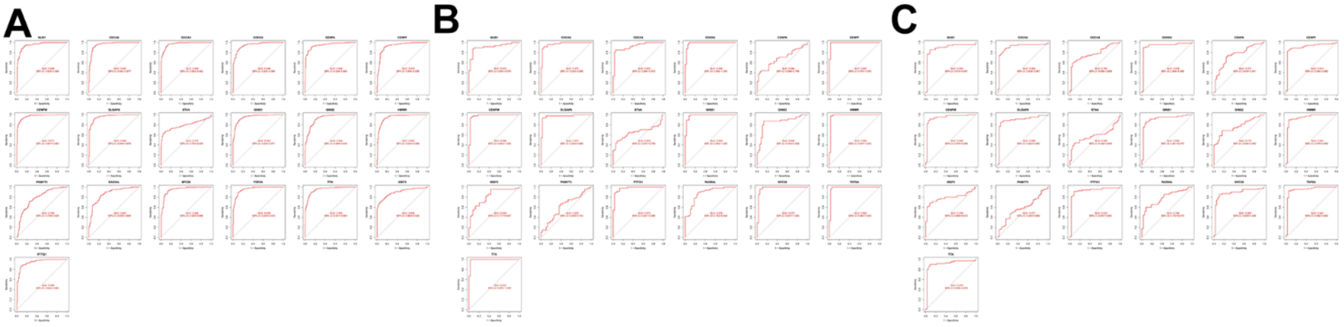

Supplementary Figure 5. ROC curves for diagnostic efficacy verification. (**A**) TCGA-LIHC (**B**) GSE45267 (**C**) GSE121248.

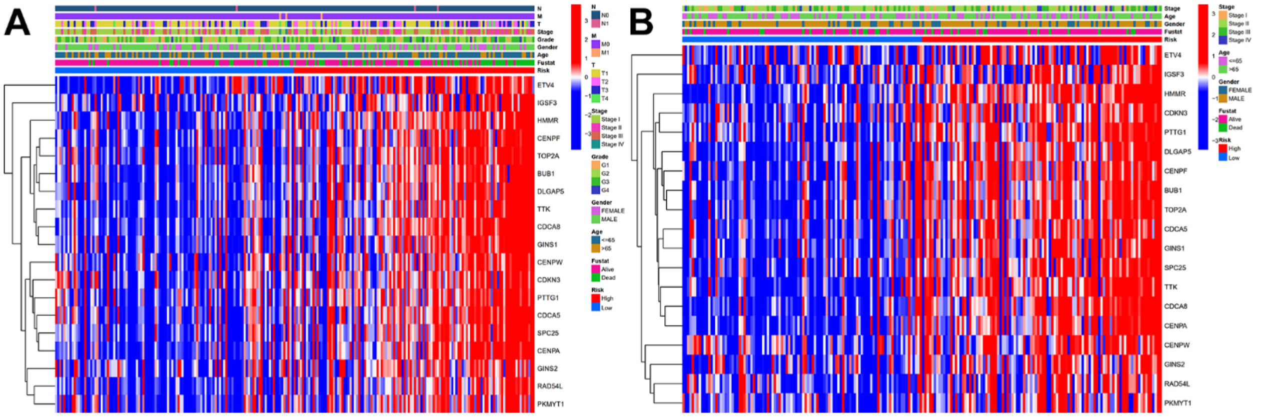

Supplementary Figure 6. Heatmap of the 19 cellular senescence-related prognosis signature and clinicopathological manifestations. (**A**) TCGA-LIHC cohort (**B**) HCCDB18 cohort.

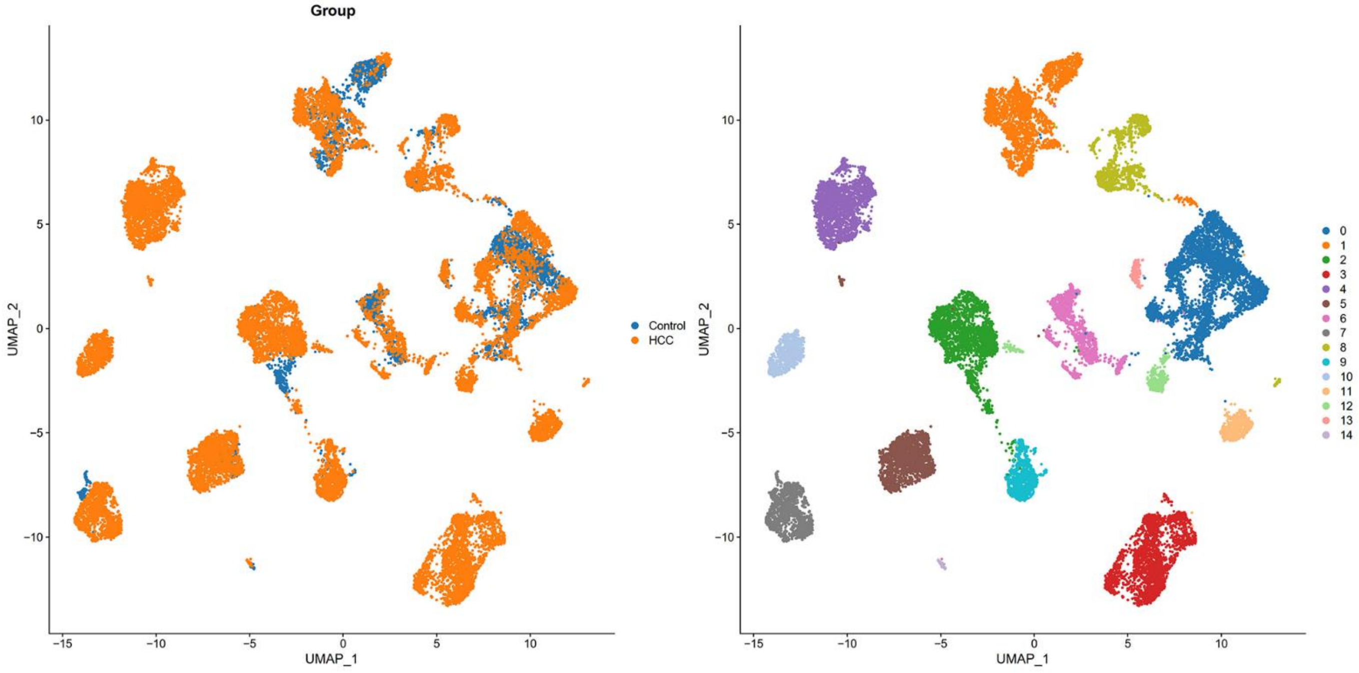

Supplementary Figure 7. Cells in non-tumor liver samples and HCC samples were classified into 14 clusters by UMAP dimension reduction.

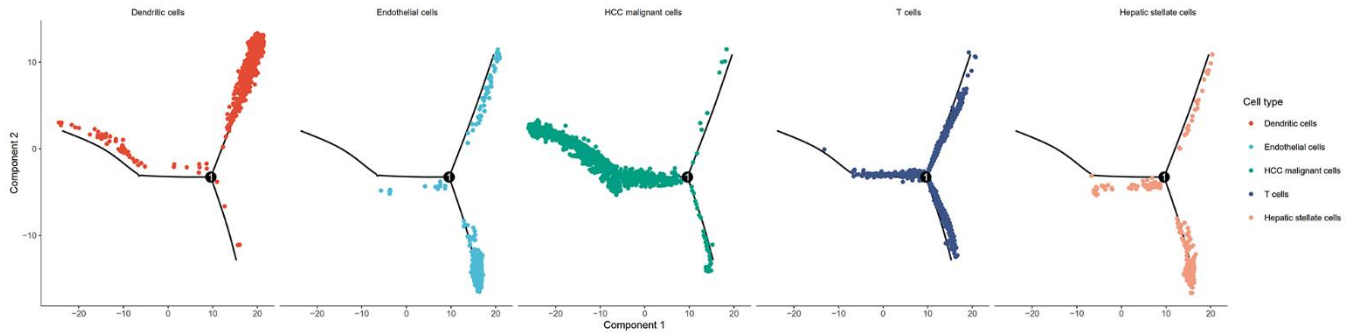

Supplementary Figure 8. The trajectory plots of each cell cluster.

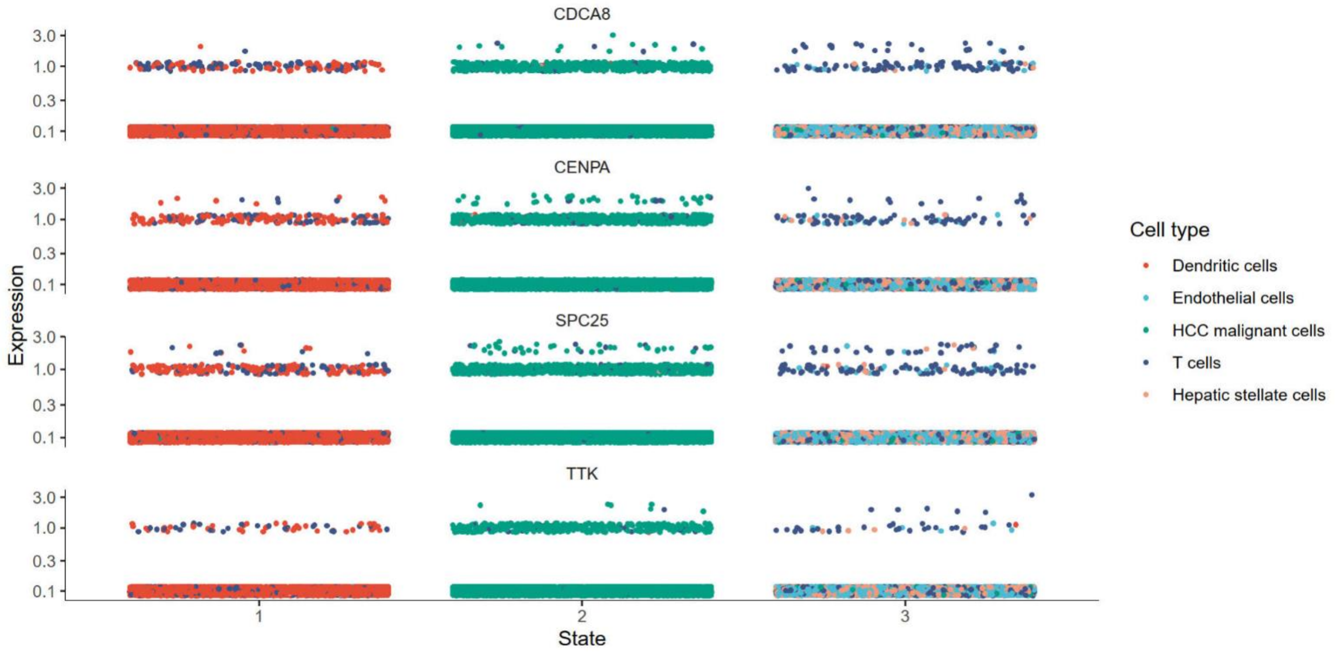

Supplementary Figure 9. The dithering plot shows the expressions of the four hub genes among five cell types in different states.
