# Supplementary material for: Cellular senescence-related gene signature as a valuable predictor of prognosis in hepatocellular carcinoma
Source: Aging (Albany NY). 2023 Apr 13;15(8):3064–93. doi: 10.18632/aging.204658 (PMC10188344; doi:10.18632/aging.204658)
Supplement: Supplementary Table 1 [file aging-15-204658-s002.pdf]

Supplementary Table 1. Clinical information of patients in the TCGA-LIHC and HCCDB18 datasets.

Variable	TCGA-LIHC	HCCDB18
Gender		
Male/Female	255/122	192/68
Age at diagnosis		
≤65/>65/NA	235/141/1	98/162
Grade		
G1/G2/G3/G4/NA	55/180/124/13/5	NA
Stage		
I/II/III/IV/NA	175/87/86/5/24	40/117/80/23
T		
T1/T2/T3/T4/NA	185/95/81/13/3	NA
M		
M0/M1/NA	272/4/101	NA
N		
N0/N1/NA	257/4/116	NA
