# Supplementary material for: Cellular senescence-related gene signature as a valuable predictor of prognosis in hepatocellular carcinoma
Source: Aging (Albany NY). 2023 Apr 13;15(8):3064–93. doi: 10.18632/aging.204658 (PMC10188344; doi:10.18632/aging.204658)
Supplement: Supplementary Table 3 [file aging-15-204658-s004.pdf]

Supplementary Table 3. Samples information of GSE45267 dataset.

Sample	Type	Age
GSM1100370	Tumor	48
GSM1100371	Tumor	41
GSM1100372	Tumor	67
GSM1100373	Tumor	41
GSM1100374	Tumor	53
GSM1100375	Tumor	47
GSM1100376	Tumor	57
GSM1100377	Tumor	51
GSM1100378	Tumor	57
GSM1100379	Tumor	60
GSM1100380	Tumor	57
GSM1100381	Tumor	55
GSM1100382	Normal	41
GSM1100383	Normal	41
GSM1100384	Normal	41
GSM1100385	Normal	51
GSM1100386	Normal	57
GSM1100387	Normal	60
GSM1100388	Normal	57
GSM1100389	Normal	68
GSM1100390	Normal	63
GSM1100391	Normal	63
GSM1100392	Normal	67
GSM1100393	Normal	68
GSM1100394	Normal	64
GSM1100395	Normal	50
GSM1100396	Normal	68
GSM1100397	Normal	51
GSM1100398	Normal	62
GSM1100399	Normal	61
GSM1100400	Normal	74
GSM1100401	Normal	46
GSM1100402	Normal	68
GSM1100403	Normal	56
GSM1100404	Normal	51
GSM1100405	Normal	51
GSM1100406	Tumor	43
GSM1100407	Tumor	62
GSM1100408	Tumor	51
GSM1100409	Tumor	68
GSM1100410	Tumor	63
GSM1100411	Tumor	68
GSM1100412	Tumor	64
GSM1100413	Tumor	41
GSM1100414	Tumor	52
GSM1100415	Tumor	50
GSM1100416	Tumor	68

GSM1100417	Tumor	62
GSM1100418	Tumor	61
GSM1100419	Tumor	52
GSM1100420	Tumor	73
GSM1100421	Tumor	61
GSM1100422	Tumor	46
GSM1100423	Tumor	65
GSM1100424	Tumor	68
GSM1100425	Tumor	51
GSM1100426	Tumor	28
GSM1100427	Tumor	30
GSM1100428	Tumor	33
GSM1100429	Tumor	36
GSM1100430	Tumor	26
GSM1100431	Tumor	39
GSM1100432	Normal	38
GSM1100433	Normal	28
GSM1100434	Normal	37
GSM1100435	Normal	37
GSM1100436	Normal	28
GSM1100437	Normal	36
GSM1100438	Normal	36
GSM1100439	Normal	37
GSM1100440	Normal	31
GSM1100441	Normal	30
GSM1100442	Normal	33
GSM1100443	Normal	32
GSM1100444	Normal	36
GSM1100445	Normal	37
GSM1100446	Normal	32
GSM1100447	Tumor	37
GSM1100448	Tumor	31
GSM1100449	Tumor	36
GSM1100450	Tumor	37
GSM1100451	Tumor	34
GSM1100452	Tumor	33
GSM1100453	Tumor	32
GSM1100454	Tumor	37
GSM1100455	Tumor	38
GSM1100456	Tumor	40
