# Supplementary material for: Cellular senescence-related gene signature as a valuable predictor of prognosis in hepatocellular carcinoma
Source: Aging (Albany NY). 2023 Apr 13;15(8):3064–93. doi: 10.18632/aging.204658 (PMC10188344; doi:10.18632/aging.204658)
Supplement: Supplementary Table 9 [file aging-15-204658-s010.pdf]

Supplementary Table 9. The immune responses in low- and high-risk groups.

Immunological response	Model	P-value	Correlation coefficient
B cell_TIMER	Senescence Score	0.0000202702295798352	0.258373290755954
T cell CD4+_TIMER	Senescence Score	0.000108400568341618	0.276193355263645
Neutrophil_TIMER	Senescence Score	4.29428339298687E-08	0.359266872808306
Macrophage_TIMER	Senescence Score	7.36486902728179E-09	0.362765093078553
Myeloid dendritic cell_TIMER	Senescence Score	5.76044404685146E-07	0.338784817248774
B cell memory_CIBERSORT	Senescence Score	0.0378522902508948	0.112089355993383
T cell CD4+ memory resting_CIBERSORT	Senescence Score	0.0175612750770366	-0.128923578822784
T cell CD4+ memory activated_CIBERSORT	Senescence Score	0.0233864281546951	0.147743753544652
T cell follicular helper_CIBERSORT	Senescence Score	0.0200831092012567	0.136804131490506
T cell regulatory (Tregs)_CIBERSORT	Senescence Score	0.000801931929368885	0.197993745095327
NK cell resting_CIBERSORT	Senescence Score	0.0439073636948704	-0.140285865065209
Monocyte_CIBERSORT	Senescence Score	0.020956636507953	-0.227470384720351
Macrophage M0_CIBERSORT	Senescence Score	0.0000325616513527694	0.262960455627498
Neutrophil_CIBERSORT	Senescence Score	0.00557075957224343	-0.132177142557853
B cell memory_CIBERSORT-ABS	Senescence Score	0.0241246554160234	0.132131610961948
B cell plasma_CIBERSORT-ABS	Senescence Score	0.0208314473996641	0.125737390349023
T cell CD4+ memory activated_CIBERSORT-ABS	Senescence Score	0.0212610117613799	0.143284248953572
T cell follicular helper_CIBERSORT-ABS	Senescence Score	0.0000507802656797722	0.155706743376464
T cell regulatory (Tregs)_CIBERSORT-ABS	Senescence Score	4.17710717662559E-07	0.15061580904037
NK cell activated_CIBERSORT-ABS	Senescence Score	0.000554593464978019	0.257548166634541
Macrophage M0_CIBERSORT-ABS	Senescence Score	9.65177541381699E-08	0.329426496419035
Macrophage M1_CIBERSORT-ABS	Senescence Score	0.0130128009570688	-0.12552569328725
Macrophage M2_CIBERSORT-ABS	Senescence Score	0.0000150217615593861	0.222457927373238
Myeloid dendritic cell resting_CIBERSORT-ABS	Senescence Score	0.0241178289648506	0.339560040235058
Neutrophil_CIBERSORT-ABS	Senescence Score	0.00124200448640288	0.192751394140667
B cell_QUANTISEQ	Senescence Score	0.0000643869176409429	0.276161027384921
Macrophage M1_QUANTISEQ	Senescence Score	0.00337453681854056	0.1598896783221
Monocyte_QUANTISEQ	Senescence Score	0.0000231614083032618	0.108902626217504
T cell CD8+_QUANTISEQ	Senescence Score	0.00199222518193981	0.16571989414694
T cell regulatory (Tregs)_QUANTISEQ	Senescence Score	0.000286306833010126	0.27318618309581
T cell_MCPCOUNTER	Senescence Score	0.000119493212173534	0.238465792530489
NK cell_MCPCOUNTER	Senescence Score	0.0422062311358533	0.116740419271637
B cell_MCPCOUNTER	Senescence Score	0.00145059540981016	0.251447153246182
Monocyte_MCPCOUNTER	Senescence Score	2.77700216856047E-07	0.221234849725056
Macrophage/Monocyte_MCPCOUNTER	Senescence Score	2.77700216856047E-07	0.271456621431428
Myeloid dendritic cell_MCPCOUNTER	Senescence Score	0.0045805073001399	0.292025625396387
Cancer associated fibroblast_MCPCOUNTER	Senescence Score	0.0425087936013699	0.15507511636032
B cell_XCELL	Senescence Score	0.0000608363256432314	0.120319182277413
T cell CD4+ memory_XCELL	Senescence Score	0.000555083730584137	0.230720862339996
T cell CD4+ central memory_XCELL	Senescence Score	0.0248439449331524	0.337129925991368
T cell CD8+ naive_XCELL	Senescence Score	0.00634942257844814	0.337129925991368
Class-switched memory B cell_XCELL	Senescence Score	0.0041932350457448	0.22906769852649

Common lymphoid progenitor_XCELL	Senescence Score	8.21845778024016E-11	0.152585860131335
Common myeloid progenitor_XCELL	Senescence Score	0.0465844913423528	0.213402788168852
Endothelial cell_XCELL	Senescence Score	1.87407397993098E-13	0.237835606872808
Granulocyte-monocyte progenitor_XCELL	Senescence Score	0.0156335953980959	-0.142477250025859
Hematopoietic stem cell_XCELL	Senescence Score	2.33714682908975E-08	-0.171024757884418
Macrophage M2_XCELL	Senescence Score	0.000704581626806011	0.373384278942444
Monocyte_XCELL	Senescence Score	0.0136449770828341	-0.130356009928635
B cell naive_XCELL	Senescence Score	0.0429814835103456	-0.445954313045994
T cell gamma delta_XCELL	Senescence Score	0.0222387582939616	-0.160291337446559
T cell CD4+ Th2_XCELL	Senescence Score	1.57099602617734E-13	-0.352018615036676
Stroma score_XCELL	Senescence Score	1.03853480503691E-09	0.121120459501573
Microenvironment score_XCELL	Senescence Score	0.0181827404380113	-0.177367742413254
B cell_EPIC	Senescence Score	0.0341078893148881	-0.115437672590154
Cancer associated fibroblast_EPIC	Senescence Score	0.0000465336670014947	0.266462663767851
Macrophage_EPIC	Senescence Score	7.06005744729839E-12	-0.405241805129521
